# When Hepatitis B Virus Meets Interferons

**DOI:** 10.3389/fmicb.2018.01611

**Published:** 2018-07-18

**Authors:** Guangyun Tan, Hongxiao Song, Fengchao Xu, Genhong Cheng

**Affiliations:** ^1^Department of Immunology, Institute of Translational Medicine, The First Hospital of Jilin University, Changchun, China; ^2^Department of Microbiology, Immunology and Molecular Genetics, University of California, Los Angeles, Los Angeles, CA, United States; ^3^Center of System Medicine, Institute of Basic Medical Sciences, Chinese Academy of Medical Sciences and Peking Union Medical College, Beijing, China; ^4^Suzhou Institute of Systems Medicine, Suzhou, China

**Keywords:** interferon, HBV, ISGs, HBV drug, cccDNA, TRIMs

## Abstract

Chronic hepatitis B virus (HBV) infection imposes a severe burden on global public health. Currently, there are no curative therapies for millions of chronic HBV-infected patients (Lok et al., 2017). Interferon (IFN; including pegylated IFN) is an approved anti-HBV drug that not only exerts direct antiviral activity, but also augments immunity against HBV infection. Through a systematic review of the literature, here we summarize and present recent progress in research regarding the interactions between IFN and HBV as well as dissect the antiviral mechanisms of IFN. We focus on inhibition of HBV replication by IFN-stimulated genes (ISGs) as well as inhibition of IFN signaling by HBV and viral proteins. Finally, we briefly discuss current IFN-based HBV treatment strategies. This review may help to better understand the mechanisms involved in the therapeutic action of IFN as well as the crosstalk between IFN and HBV, and facilitate the development of both direct-acting and immunology-based new HBV drugs.

## Introduction

More than 50 years have passed since the identification of human hepatitis B virions, initially named “Dane particles,” in the late 1960s; and hepatitis B virus (HBV) infection remains a serious global health problem (Hirschman et al., 1969; Cossart and Field, 1970; Dane et al., 1970; Zanen-Lim, 1976; Glebe, 2007). Three-quarters of the world’s population live in HBV endemic regions, and one-third of them have been exposed to HBV. Despite the implementation of HBV vaccination for nearly 30 years, 240 million of 2 billion HBV-infected people worldwide have become chronically infected. Approximately 39 million chronic HBV infections occur in the region of Southeast Asia (Childs et al., 2018), and there are an estimated 100 million HBV surface antigen (HBsAg)-positive people in both China and Africa who are at high risk for developing liver cirrhosis and hepatocellular carcinoma (HCC) (El-Serag and Rudolph, 2007; Levrero and Zucman-Rossi, 2016). The World Health Organization estimates that there were 1.34 million HBV infection-related deaths in 2015, which is comparable to the annual deaths caused by tuberculosis and is higher than HIV-related mortality (Breakwell et al., 2017).

Interferons (IFNs) are a group of signaling proteins made and released by host cells in response to various pathogens, including bacteria, viruses, and parasites (De Andrea et al., 2002; Lee and Baldridge, 2017). Based on the receptor type to which they bind, human IFNs are classified into type I, II, and III (Isaacs and Lindenmann, 1957; Wheelock, 1965; Sheppard et al., 2003; Vilcek, 2006; **Table 1**). IFN-α and pegylated IFN-α have been approved for the treatment of chronic hepatitis B (De Andrea et al., 2002; Liang et al., 2015). In addition, IFN-α has been reported to inhibit HBV replication in *in vitro* systems (Wieland et al., 2003; Pollicino et al., 2013; Lin and Young, 2014). HBV infection can activate the innate immune response in the liver and trigger the local production of type I or type III IFN (Wieland et al., 2004; Shlomai et al., 2014b; Sato et al., 2015). However, it also has been reported that the host’s response to HBV infection does not induce production of either type I or III IFN (Cheng et al., 2017).

**Table 1:** Current classification of human interferons.

Properties (Reference)	Year of discovery	Family members	Receptor	IFN-producing cells	Signaling	Specific genes
Type I IFN (Isaacs and Lindenmann, 1987; Bekisz et al., 2004; Liu et al., 2012)	1957	IFN-α, IFN-β, IFN-ω, IFN-δ, IFN-τ	IFNαR1/2 subunits	All nucleated cells	TYK2, JAK1, STATs (mainly STAT1/2), bind to ISRE/GAS	Mx1, IL-10, MMP13, CCL4, etc.
Type II IFN (Wheelock, 1965; Liu et al., 2012; Castro et al., 2018)	1965	IFN-γ	IFN-γR1/2 subunits	Activated lymphocytes (T and B cells), NK and NKT cells, APCs	JAK1/2, STAT1, bind to GAS/ISRE	TRIM16, Gpr18, etc.
Type III IFN (Sheppard et al., 2003; Wack et al., 2015; Obajemu et al., 2017; Xu et al., 2018)	2003	IL-29/IFN-λ1, IL-28A/IFN-λ2, IL-28B/IFN-λ3, IFN-λ4	IL-28R, IL-10Rβ	All nucleated cells, mainly mDCs, pDCs, and epithelial cells	TYK2, JAK1, ISGF3, bind to ISRE	CBFβ, etc.

*IFN-αR, IFN-α receptor; IFN-γR, IFN-γ receptor; IL-28R, interleukin-28 receptor; IL-10Rβ, interleukin-10 receptor β; TYK, tyrosine kinase; JAK, Janus kinase; STAT, signal transducer and activator of transcription protein; ISRE, interferon-sensitive response element; GAS, gamma-activated site; ISGF3, STAT1-STAT2-IRF9 complex; mDCs, myeloid dendritic cells; pDCs, plasmacytoid dendritic cells; APCs, antigen-presenting cells; Mx1, interferon-induced GTP-binding protein Mx1; MMP13, matrix metalloproteinase 13; CCL4, chemokine (C-C motif) ligand 4; TRIM16, tripartite motif-containing protein 16; Gpr18, G protein-coupled receptor 18; CBFβ, core-binding factor subunit β.*

Many IFN-stimulated genes (ISGs) have been shown to inhibit HBV infection and replication through different pathways, and many studies have tried to understand why IFN signaling is weak in an HBV-infected liver. In this review, we discuss the crosstalk between IFN and HBV based on the results generated in our lab and those published by others, which may shed new light on the interaction between IFN and HBV as well as provide insight into new HBV drug discovery and development. This review consists of three sections: (1) the HBV life cycle and the classical IFN induction pathway in response to viral infection; (2) a systematic discussion of the crosstalk between IFN and HBV, with a focus on two-way inhibition: the inhibition of HBV infection and replication by ISGs as well as the inhibition of IFN signaling by HBV and HBV proteins; and (3) a brief discussion of the current IFN-based HBV treatment strategies.

## HBV Genome Organization and Life Cycle

Hepatitis B virus belongs to the *Hepadnaviridae* family and contains a 3.2-kb partially double-stranded DNA (Evans and Seeger, 2007). Once entered into blood circulation, HBV binds to its receptor, currently known as sodium taurocholate co-transporting polypeptide (NTCP), a multiple transmembrane transporter predominantly expressed in hepatic cells (Yan et al., 2012), resulting in viral entry. In the cytoplasm, the virus is caught by the endosomes. In response to endosome maturation, the viral envelope fuses with the endosome membrane and releases the free nucleocapsid into the cytoplasm (Hayes et al., 2016). The nucleocapsid is translocated to the nucleus where relaxed circular DNA is released. And under the help of cellular replicative machinery (Levrero et al., 2009; Koniger et al., 2014), the relaxed circular DNA is converted into a covalently closed circular DNA (cccDNA), which further interacts with histone proteins to form a minichromosome. HBV synthesizes the capsid in the cytoplasm. It appears that cccDNA in infected cells is difficult to eliminate (Tong and Revill, 2016).

**FIGURE 1:**
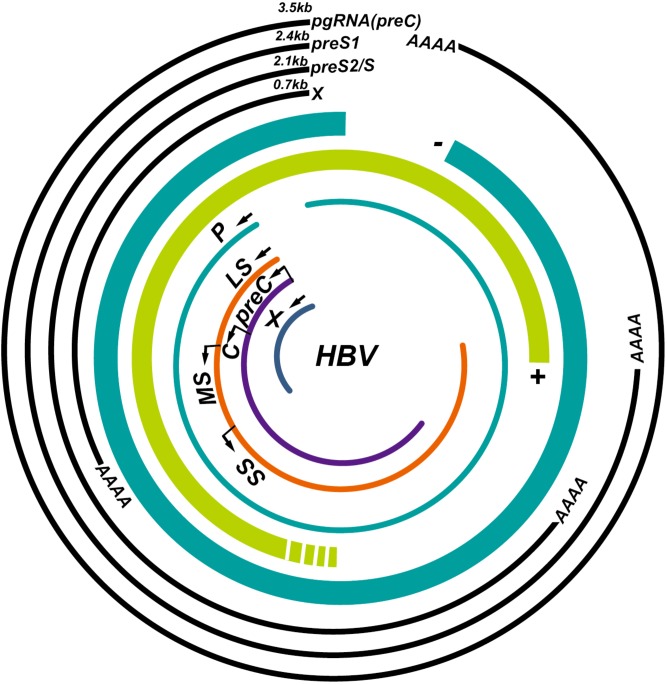
Illustration of the hepatitis B virus (HBV) genome and its overlapping open reading frames and transcripts. HBV is a partially double-stranded DNA virus that transcribes four major transcripts: pgRNA (3.5 kb), preS1 (2.4 kb), preS2/S (2.1 kb), and X (0.7 kb) and synthesizes seven proteins: P, polymerase; LS, large S; MS, middle S; SS, small S; preC, pre-Core; C, core; X, HBx.

**FIGURE 2:**
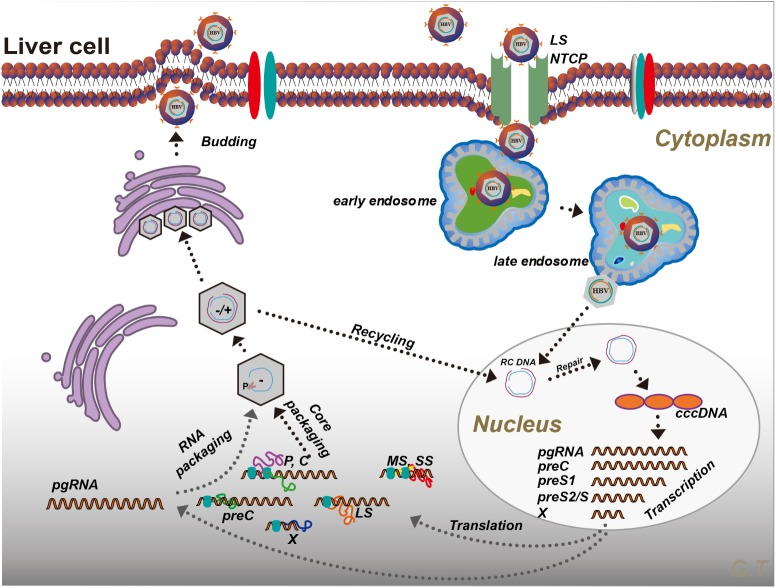
Hepatitis B virus life cycle. Please see the text for details. P, polymerase; LS, large S; MS, middle S; SS, small S; preC, pre-Core; C, core; X, HBx; RC DNA, relaxed circular DNA; NTCP, sodium taurocholate co-transporting polypeptide; cccDNA, covalently closed circular DNA.

The HBV genome contains four overlapping open reading frames (S, C, P, and X) (**Figure 1**). HBV cccDNA functions as a transcription template, encoding four major viral RNA species: pregenomic RNA (pgRNA, 3.5 kb), preS1 HBs RNA (2.4 kb), preS2/S HBs RNA (2.1 kb), and HBV X protein RNA (HBx, 0.7 kb) (**Figure 1**; Tuttleman et al., 1986; Newbold et al., 1995). The viral RNA transcription is initiated by activating four promoters (the core, pre-S1, pre-S2/S, and X promoters) with host-specific transcriptional factors such as hepatocyte nuclear factors (NFs) and epigenetic modifications (Pollicino et al., 2006; Kim et al., 2016). The HBV genome also contains two enhancer sites (En I and En II) for upregulation of transcription (Hu and Siddiqui, 1991). Viral mRNAs are then transferred to the cytoplasm where they function as templates either for the synthesis of viral proteins or for the synthesis of minus-strand DNA (reverse transcription) (Shlomai et al., 2014a). The HBV mRNAs encode seven proteins depending on the locations of the initiation codon in the RNA transcripts: preC mRNA for HBV e antigen (HBeAg) (3.5 kb), pgRNA for HBV c antigen (HBcAg) and Pol (3.5 kb), preS1 for large S (2.4 kb), preS2/S mRNAs for middle and small surface proteins (MS and HBsAg, 2.1 kb), and HBx mRNA for HBxAg (0.7 kb) (**Figure 1**; Liang, 2009). Once viral protein synthesis reaches a certain level in the cytoplasm, pgRNA is selectively packaged into the “HBcAg house” along with Pol protein, which form the immature nucleocapsid, and then pgRNA is reversely transcribed to the minus-strand DNA catalyzed by the reverse transcriptase domain of Pol. A variable length of plus-strand HBV DNA is synthesized using the minus strand as the template to generate the HBV genome. The mature HBV capsid is either enveloped with HBsAg and released from hepatocytes or recycled to the nucleus for amplification of the cccDNA pool (**Figure 2**; Rehermann and Nascimbeni, 2005).

## Classical IFN and ISG Induction Pathways in Response to Viral Infection

**FIGURE 3:**
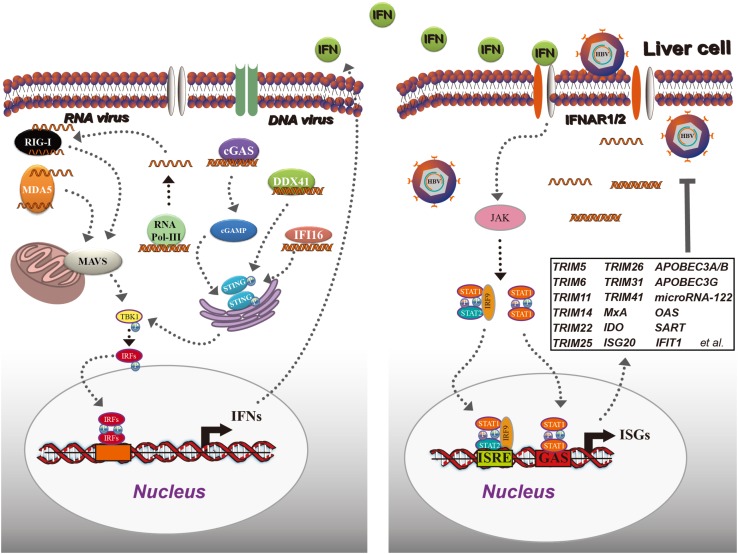
Classical interferon (IFN) and IFN-stimulated gene (ISG) induction in response to viral infection. In the cytoplasm, viral DNA is recognized by cGAS, DDX41, or IFI16, leading to the activation of STING, which further recruits TBK1 for IRF activation and translocates to the nucleus to initiate IFN production. Viral RNA is detected by RIG-I and MDA5, and recruits another adaptor protein: MAVS (IPS-1), resulting in TBK1 activation, IRF phosphorylation, and IFN induction. Then, IFNs are secreted from the cells and bind to IFNAR1/2, leading to JAK/STAT activation. The activated ISGF3 trimer or STAT1 dimer bind to the ISRE or GAS sequence and promote the expression of ISGs. ISGs inhibit HBV as shown in the *black box*. cGAS, cyclic GMP-AMP synthase; DDX41, DEAD-box protein 41; IFI16, γ-IFN-inducible protein 16; STING, stimulator of IFN genes; TBK1, TANK-binding kinase 1; IFNAR1, type I IFN receptor 1; JAK, Janus tyrosine kinase; STAT, signal transducer and activator of transcription.

In response to viral infection, IFN production can be efficiently induced in many types of cells by the activation of pattern-recognition receptors. Activation of type I IFN family genes constitutes one of the earliest transcriptional responses, perhaps representing the most important innate immune response to viral infection. Most nucleated vertebrate cells are able to both produce and respond to type I IFN. The detection of intracellular microbial DNA and RNA in macrophages and/or infected cells is critical to elicit an appropriate innate immune response. During viral infection, virus-derived nucleic acids (both RNA and DNA) are mainly sensed by certain cytosolic pattern-recognition receptors. For instance, viral RNA is recognized by retinoic acid inducible gene I (RIG-I)-like receptors, including RIG-I (Pichlmair et al., 2006; Myong et al., 2009) and melanoma differentiation associated protein 5 (Takeuchi and Akira, 2008, 2009), leading to its association with the mitochondrial antiviral-signaling protein (MAVS). DNA is sensed by DEAD-box protein (DDX) 41, cyclic GMP-AMP synthase (cGAS), and γ-IFN-inducible protein 16 (IFI16) (Unterholzner et al., 2010; Zhang et al., 2011; Sun et al., 2013; Bhat and Fitzgerald, 2014), all of which lead to the activation of stimulator of IFN genes (STING). Both MAVS recruitment and STING activation after RNA or DNA viral infection lead to TANK-binding kinase 1 (TBK1) phosphorylation and activation of IFN regulatory factors (IRFs), which are primarily responsible for IFN production (Sato et al., 2000). Cytosolic RNA polymerase III is specialized to detect foreign and aberrant DNA, which is converted into RNA. RNA synthesized in the cytosol contains 5′-triphosphate, which can be detected by RIG-I. Therefore, RNA polymerase III can act as a DNA sensor that triggers the innate immune response through the RIG-I pathway (Chiu et al., 2009; **Figure 3**).

The antiviral function of type I IFN is cascaded via binding to type I IFN receptor (IFNAR1), activating the Janus tyrosine kinase (JAK)/signal transducer and activator of transcription (STAT) pathway, and subsequently inducing approximately 300 ISGs (Ivashkiv and Donlin, 2014). Different ISGs can inhibit different stages of the viral life cycle (Liu et al., 2013; Chen et al., 2014). The HBV replication steps that can be inhibited by ISGs are illutrstated in **Figure 3**.

## How HBV Interacts with IFN Induction and Signaling

As reported previously, the lack of a functional innate DNA-sensing pathway in hepatocytes allows HBV to establish infection and to replicate (Thomsen et al., 2016). As a consequence of co-evolution, viruses have acquired the ability to counter the antiviral response induced by type I IFN. First, some host factors that are pro-HBV replication or antagonize the innate immunity are induced after HBV infection. For instance, NF-κB essential modulator (NEMO) has an essential role in the production of IFN-β by phosphorylating and activating IFN regulatory factor 3 (IRF3) in a polyubiquitin-dependent manner (Clark et al., 2013); and rubicon, a virus-induced protein, has been reported to bind to NEMO, which inhibits the ubiquitination of NEMO and further inhibits type I IFN production (Wan et al., 2017). In addition, HBV-induced parkin can recruit a linear ubiquitin assembly complex (LUBAC) to mitochondria and modulate K-63 and the linear ubiquitin chain on the MAVS signalosome, which abrogates IFN-β synthesis (Khan et al., 2016). These two reports clearly indicate the mechanism of HBV in regulating some of the critical factors in IFN production. Moreover, HBV is also reported to inhibit IFN signaling by regulating IFNAR1, and HBV-activated collagen triple helix repeat containing 1 (CTHRC1) reduces IFNAR1/2 expression and downregulates the activity of type I IFN (Bai et al., 2015). However, how IFNAR1/2 is reduced has not been clarified. Interestingly, another report has shown that HBV can induce matrix metalloproteinase 9 (MMP9), which binds to IFNAR1, promotes its degradation via ubiquitination, and suppresses IFN/JAK/STAT signaling (Chen et al., 2017).

Second, HBV or viral proteins also have been clearly reported to inhibit IFN production. Major vault protein (MVP) is a novel virus-induced cellular protein that upregulates type I IFN production. The middle domain of MVP (amino acid residues 310–620) is essential for MyD88 binding; interestingly, both HBsAg and HBeAg have been shown to suppress the interaction between MVP and MyD88 (Kawai et al., 2004), which leads to limiting the downstream IFN signaling (Liu S. et al., 2015). In addition, HBV Pol has been shown to interact with STING and to decrease the Lys63-linked polyubiquitination of STING dramatically, resulting in inhibiting the activation of STING-stimulated IRF3 and decreasing the induction of IFN-β (Liu Y. et al., 2015). Moreover, Pol has been reported to evade the innate immune response in the early phase of infection, thus interrupting the interaction between the inhibitor of NF-κB kinase (IKK) 𝜀 and DDX3 DEAD box RNA helicase as well as inhibit TBK1/IKK𝜀 activity (Wang and Ryu, 2010). Interestingly, HBV viral particles have appeared to readily inhibit the innate immune response through virion/cell interactions (Luangsay et al., 2015), which might partially explain the “stealthy” characteristics of HBV. *In vitro*, upon infection of primary hepatocytes with HBV, both type I and III IFN transcripts were suppressed to 10% of the levels observed in uninfected cultures, suggesting an active role for HBV in suppressing innate immune activation from the side (Ortega-Prieto et al., 2018).

**FIGURE 4:**
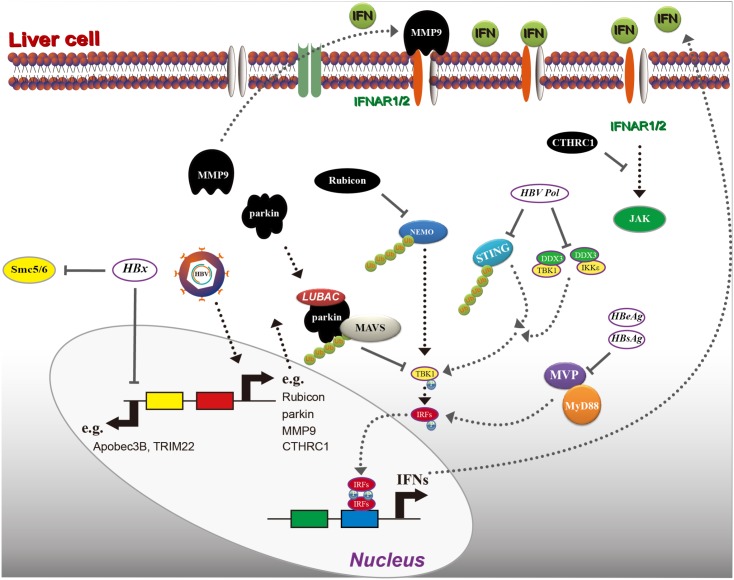
Hepatitis B virus interrupts interferon (IFN) signaling. As indicated, HBV induces several host factors, including rubicon, parkin, MMP9, and CTHRC1. These proteins inhibit IFN production in a different mechanism, and HBV proteins including HBx, Pol (polymerase), HBsAg, and HBeAg can also block IFN induction or directly inhibit ISG induction. Please see the text for details. MVP, major vault protein; MMP9, matrix metalloproteinase 9; NEMO, nuclear factor-κB essential modulator; LUBAC, linear ubiquitin assembly complex; RIG-I, retinoic acid inducible gene I; MAVS, mitochondrial antiviral-signaling protein.

Another viral protein is HBx, which is a 17-kDa protein, consisting of an *N*-terminal negative regulatory domain and a *C*-terminal transactivation or co-activation domain (Gong et al., 2013). HBx may function as a transactivator and appears to regulate the expression of cellular genes; in addition, it is implicated in the development of HCC (Zhang et al., 2017). HBx interacts with several cellular proteins and impacts HBV replication through these interactions. The best-characterized HBx binding partner is damage-specific DNA binding protein 1 (DDB1), and the interaction between HBx and DDB1 is essential for HBV replication (Hodgson et al., 2012). HBx induces cytokine signaling 3 and protein phosphatase 2A suppression, impairing IFN signaling (Tsunematsu et al., 2017). HBx has been reported to elevate MSL2 expression and degrade APOBEC3B, leading to cccDNA accumulation and hepatocarcinogenesis (Gao et al., 2017). HBx also has been shown to induce a single CpG methylation in the 5′-UTR of the tripartite motif 22 (TRIM22) gene and inhibit IFN-induced TRIM22 expression (Lim et al., 2017). These reports demonstrate that HBx has a negative impact on interrupting IFN signaling and producing both IFN and ISGs. Several reports have shown that HBx targets the structural maintenance of chromosomes (SMC)5/6 complex to enhance HBV gene expression from episomal cccDNA (Murphy et al., 2016; Livingston et al., 2017). It has been reported that the SMC complex is not induced by IFN. However, SMC5/6 forms the foundation of a multi-subunit DNA repair complex and is a restriction factor that selectively blocks extrachromosomal DNA transcription (Uhlmann, 2016). Moreover, it has been demonstrated that HBx binds to the DDB1 subunit of the DDB1-containing E3 ubiquitin ligase and forms an HBx-DDB1-cullin 4-ROC1 E3 ligase complex for ubiquitin-mediated degradation of SMC5/6 complexes (Decorsiere et al., 2016), which lift the restriction of HBV gene expression from episomal cccDNA. A disruption of the interaction between HBx and host proteins might increase the efficiency of IFN treatment (**Figure 4**).

Hepatitis B virus DNA has been reported to be recognized by cGAS, resulting in activation of the cGAS-STING pathway, which can suppress HBV replication by inducing ISG56 (IFIT1) (Dansako et al., 2016). However, IFN induction is not presented in this report. In addition, HBV pgRNA can be recognized by RIG-I, leading to induction of type III but not type I IFN (Sato et al., 2015). HBV replication generates both DNA and RNA species; therefore, many more studies are required to elucidate the complicated mechanisms involved in the immune response to HBV infection.

Hepatitis B virus pgRNA represents another target by the host immunity as it is recognized by RIG-I, thus eliciting type III IFN induction (Sato et al., 2015), which can also inhibit HBV replication (Hong et al., 2007; Pagliaccetti et al., 2010; Tian et al., 2014). In our recent study, we have found that type III IFN inhibits HBV replication through inducing core-binding factor beta (CBFβ). It is known that CBFβ suppresses the formation of the HBx-DDB1-SMC5/6 complex, which blocks HBx transcriptional activity (Xu et al., 2018).

Taken together, there is complicated crosstalk between HBV and IFN/ISGs. Therefore, a thorough understanding of the mechanisms responsible for the HBV-mediated inhibition of IFN is needed for designing and developing immunology-based HBV therapy.

## How ISGs Inhibit HBV Replication

Many ISGs are known to inhibit HBV replication. One of them is the *APOBEC* gene, which encodes a family of cytidine deaminases that edit DNA and/or RNA bases by deaminating cytidine and converting it to uridine. A total of 11 members of this family are encoded by the human genome (Vieira and Soares, 2013), among which *APOBEC3A/B* has been shown to be induced by IFN, resulting in cytidine deamination, apurinic/apyrimidinic site formation, and finally cccDNA degradation that prevents HBV reactivation in HBV-infected cells (Lucifora et al., 2014). We also have reported that APOBEC3G inhibits HBV replication and that IFN-induced APOBEC3G is STAT3 but not STAT1 dependent. However, A3G expression is reduced in HBV-infected patients, which might due to the inhibition of STAT3 by HBsAg (Xu et al., 2016).

In addition, ISG20 inhibits HBV replication through directly binding to the epsilon stem-loop structure of pgRNA (Liu et al., 2017). Indoleamine-pyrrole 2,3-dioxygenase, an IFN-induced enzyme catalyzing tryptophan degradation, has been found to reduce the intracellular HBV DNA level efficiently by tryptophan deprivation without altering the steady-state level of viral RNA (Mao et al., 2011). Myxovirus resistance protein 1 (MxA) interacts with the HBV core protein, which might interfere with capsid assembly (Li et al., 2012). The 2′,5′-linked oligoadenylate is synthesized by a family of enzymes named oligoadenylate synthetases, a type I IFN-inducible gene. RNase L binds to 2′,5′-linked oligoadenylate to activate it, thus inhibiting HBV RNAs (Park et al., 2014). In addition, squamous cell carcinoma antigen recognized by T cells (SART) is involved in regulating ISG expression against HBV infection after IFN treatment (Li et al., 2016). Interestingly, miR-122 also has been reported to be induced by IFN, thus inhibiting HBV replication by targeting suppressor of cytokine signaling 3, a molecule contributing to inactivation of the IFN signaling pathway (Gao et al., 2015; **Figure 3**).

In recent years, several TRIM-containing family members, which are also categorized as ISGs, have been found to be important in regulating IFN signaling and inhibiting HBV replication (Zhang et al., 2013). They comprise a really interesting new gene (RING) domain, one or two B-box domains, and an associated coiled-coil domain in the amino-terminal region and share RING finger E3 ubiquitin ligase activity (Torok and Etkin, 2001; Ozato et al., 2008; Rajsbaum et al., 2014). As reported by us and others, TRIM expression is induced in response to IFN stimulation and is required to control viral infections (Uchil et al., 2013; Versteeg et al., 2013; Liu et al., 2016; Tan G. et al., 2017). We have found that TRIM25 expression is elevated under the induction of type I IFN in an interleukin-27-dependent manner, thus inhibiting HBV replication through augmenting IFN production (Tan G. et al., 2017). TRIM22 also has been shown to inhibit HBV core promoter activity (Gao et al., 2009). Among the TRIMs, TRIM14 has recently emerged as an attractive gene that regulates IFN signaling. TRIM14 recruits ubiquitin-specific protease 14 to cleave the lysine (Lys) 48-linked ubiquitin chains of cGAS at Lys414 and facilitates the activation of type I IFN signaling, thus inhibiting p62-mediated autophagic degradation of cGAS (Chen et al., 2016). TRIM14 undergoes Lys-63-linked polyubiquitination at Lys-365 and recruits the NEMO to the MAVS signalosome, activating both the IRF3 and NF-κB pathways, which enhance the innate immune response (Zhou et al., 2014). In addition, RIG-I-mediated innate antiviral immunity has been reported to require TRIM14 to form a Werner helicase interacting protein–TRIM14–phosphoprotein phosphatase 6C mitochondrial signalosome (Tan P. et al., 2017). TRIM31 also has been shown to inhibit HBV replication and regulate IFN signaling. Mechanistically, the interaction between TRIM31 and MAVS catalyzes the Lys63 (K63)-linked polyubiquitination of Lys10, Lys311, and Lys461 on MAVS, which forms prion-like aggregates of MAVS in response to viral infection (Liu et al., 2016). In addition, additional TRIMs (e.g., TRIM56, Tsuchida et al., 2010; Wang et al., 2011; Shen et al., 2012 and TRIM38, Hu et al., 2016), potential regulators of HBV replication, have been shown to regulate IFN production (**Figure 3**). TRIMs might be potential targets for HBV therapy.

## Rational for IFN-Based Treatment of Chronic Hepatitis B

Interferon-α has been used for treating chronic hepatitis B for over two decades. Three guidelines are recommended by the American Association for the Study of Liver Diseases (AASLD): (1) risk for virological relapse, hepatic decompensation, liver cancer, and death; (2) financial concerns; and (3) preference of the patient (Ghany, 2017). Because of parenteral administration, severe side effects, and less effective inhibition of HBV DNA replication, IFN-α is not a first-line drug recommended by the guidelines, nor has it been widely used in the clinic (European Association For The Study Of The Liver, 2012). Injectable formulations of pegylated IFN-α (peg-IFN-α-2a and α-2b) are available for HBV therapy (Tang et al., 2018). peg-IFN has a longer half-life compared with standard IFN, so less frequent dosing is required and more sustained viral suppression has been shown in clinical trials compared with standard IFN (Cooksley et al., 2003). The injection of pegylated IFN-α is administered once a week in a dose of 180 μg for 48 weeks (Liaw et al., 2011). Its use is limited by adverse effects including flu-like symptoms, thyroid dysfunction, gastrointestinal symptoms, neutropenia, and so on (Marcellin et al., 2004; Janssen et al., 2005; Lau et al., 2005). According to the three major liver societies, the AASLD, the European Association for the Study of the Liver (EASL), and the Asian Pacific Association for the Study of the Liver (APASL), the IFN-α treatment guidelines are all similar (reviewed in Sato et al., 2015).

Currently, 7 therapies are approved, including standard and peg-IFN, and 5 nucleos(t)ide analogs (NAs) (NUCs: ADV, adefovir; ETV, entecavir; LMV, lamivudine; TBV, telbivudine; TDF, tenofovir), in which Peg-IFN, ETV, and TDF was recommended by the guidelines of major liver associations (Lok and McMahon, 2009; European Association For The Study Of The Liver, 2012; Liaw et al., 2012). Despite the adverse effects, IFN shows a better efficacy in inducing higher percentages of HBeAg loss as well as HBsAg loss compared to nucleoside or NAs (Konerman and Lok, 2016). Several reports showed that the outcome of peg-IFN combining with LMV treatment was similar compared to peg-IFN alone (Marcellin et al., 2004; Janssen et al., 2005; Lau et al., 2005; Buster et al., 2008). It was also reported that TDF plus peg-IFN treatment increased the loss of HBsAg than those receiving TDF or peg-IFN alone (Marcellin et al., 2016). Peg-IFN-α-2b combination with ADV therapy inhibited viral productivity by 99% and subsequent ADV monotherapy by 76%, while with only a small number of patients involved (24 patients), this clinical trial is limited (Lutgehetmann et al., 2008). Switch to TBV after 24 weeks of Peg-IFNα-2a therapy was shown to promote HBeAg seroconversion that merits investigation in HBeAg-positive CHB patients (Luo et al., 2017). Sequential approaches with peg-IFN and NUCs were also tested, however, no significant benefit was noted (Xie et al., 2014; Brouwer et al., 2015). It was reported that patients started to resistant to NUCs after long term therapy, interestingly, after subcutaneous peg-IFN add-on treatment, anti-HBs antibody appeared, which suggested peg-IFN might benefit HBsAg seroconversion (Ouzan et al., 2013; Stanzione et al., 2016). And peg-IFN monotherapy showed greater advantages in HBeAg seroconversion compared to peg-IFN-LMV combination or LMV monotherapy (32 and 27% vs. 19%) (Lau et al., 2005). The combination of high-dose peg-IFN and NAs resulted in sustained loss of serum HBV DNA and other HBV markers in an immunodeficient mouse model (Uchida et al., 2017), suggesting that HBV replication can be effectively inhibited by antivirals in the absence of a cellular immune response. A recombinant human serum albumin (rHSA)-IFN-α2a fusion protein is under development and aims to extend the *in vivo* half-life of IFN-α. The data from clinical trials reveal that rHSA/IFNα2a is well tolerated and effective at inhibiting HBV DNA (Ding et al., 2017).

Interferon treatment stayed a good alternative for people with a good prognosis (young age, low viral load, and female gender) (Sokal et al., 1998; Roberts, 2000). And due to different HBV genotype, HBeAg positive or negative stage of liver disease Peg-IFN therapy shows different response and outcome (Konerman and Lok, 2016), therefore, precision treatment should be created for every individual patient.

Recently, a combination therapy of IFN-λ with entecavir was assessed for its efficacy in treating chronic hepatitis B patients, and the trial data showed a more profound decrease in the levels of HBV DNA, HBsAg, and HBeAg compared with entecavir monotherapy (European Association For The Study Of The Liver, 2012; Phillips et al., 2017). Since only 13 patients were involved in this clinical trial, the results need to be further confirmed with many more chronic hepatitis B patient samples. Despite the severe side effects, the NAs may induce IFN-λ3 production, which triggers the expression of ISGs and reduces the HBsAg level (Murata et al., 2018). If we can clarify the exact inhibition mechanism involved in this process, a cure for HBV infection will be just around the corner.

GS-9620, a toll-like receptor 7 agonist, which was first evaluated in HBV-infected chimpanzees in 2013 (Lanford et al., 2013), has demonstrated a prolonged inhibition of HBV replication via a type I IFN-dependent mechanism (Niu et al., 2017). In combination with a NA, GS-9620 appears to increase T-cell and natural killer cell responses and to reduce the ability of natural killer cells to suppress T cells in chronic hepatitis B patients, raising the possibility that GS-9620 might potentially be used as an adjunct drug in HBV treatment to augment the immune response to HBV (Boni et al., 2018). However, comparing the data from chimpanzees and woodchuck models (Lanford et al., 2013; Menne et al., 2015), a much lower efficacy of GS-9620 in clinical trials in humans has been reported, since no significant effect was shown on serum HBsAg levels, tempering the previous expectation (Agarwal et al., 2018; Boni et al., 2018). The reason for this finding might be that the animals received higher doses (in terms of concentration and/or frequency) than those used in CHB patients.

Hepatitis B virus cccDNA resides as an episomal template in nuclei of infected hepatocytes. Although the average cccDNA copy number per cell is low (10 copies in a duck liver cell and 0.1–1 copies in a human liver cell) (Zhang et al., 2003; Volz et al., 2013), persistent or residual cccDNA is the reason why recurrent HBV infection occurs in resolved patients. A complete cure of HBV infection is a functional cure plus cccDNA elimination (Durantel, 2017). The impact of IFN treatment on cccDNA is a subject for debate. For instance, in some reports, cccDNA remains at a similar level in IFN-treated primary human hepatocytes (Niu et al., 2017; Shen et al., 2017; Mutz et al., 2018). However, other reports have shown a significant decrease in the cccDNA level after IFN treatment (Chuaypen et al., 2017; Mu et al., 2017). In addition, IFN-α has been shown to repress HBV cccDNA transcriptional activity by an epigenetic modification (Belloni et al., 2012). Moreover, a novel site-specific PEGylated recombinant human IFN-β (TRK-560) demonstrates a stronger antiviral potency by inducing a higher expression of ISGs and a stronger stimulation of immune cell chemotaxis compared with PEG-IFN-α2a. Furthermore, the reduction of cccDNA under TRK-560 treatment *in vivo* was significantly higher than that by PEG-IFN-α2a treatment (Tsuge et al., 2017). However, the cccDNA synthetic process involves several host enzymes that could be difficult to target as they are also required for other biological functions. Eradication of HBV from an infected liver requires elimination of cccDNA (Nassal, 2015), which appears to be unrealistic at this moment.

## Conclusion and Future Outlook

Evolution has equipped the human immune system with effective strategies to defend against viral infections. IFN production and signaling represent the most studied as well as the earliest immune response. Upon HBV infection, both HBV DNA and pgRNA can be recognized by both a DNA sensor (cGAS) and an RNA sensor (RIG-I). However, only type III but not type I IFN is induced in infected hepatocytes, which might reflect a lack of or inactive DNA sensing pathways in hepatocytes (Cheng et al., 2017). IFN-α has demonstrated the ability to reduce the levels of HBV DNA, HBsAg, and cccDNA efficiently. Specific and efficient activation of the IFN pathway in HBV patients might represent one treatment strategy to eradicate HBV from an infected liver. However, the challenge is that the antiviral function of IFN is compromised by the presence of HBV. As discussed above, most crosstalk between IFN and HBV is mediated by the interactions between ISG proteins and HBV proteins. For example, rubicon binds to NEMO, MMP9 binds to IFNAR, Pol interacts with STING, parkin recruits LUBAC, and HBx targets SMC5/6. All these protein interactions are deleterious to IFN signaling and benefit HBV infection. Therefore, small molecules designed to interfere with these interactions may interrupt the inhibition of IFN signaling by HBV and viral products.

## Author Contributions

GT planned the research and wrote the paper. HS and FX gave suggestions and revised the manuscript. GC conceived the research project and revised the manuscript.

## Conflict of Interest Statement

The authors declare that the research was conducted in the absence of any commercial or financial relationships that could be construed as a potential conflict of interest.
